# Dr. W. J. McGee

**Published:** 1912-10

**Authors:** 


Dr. W. J. McGee, the distinguished geologist and ethnologist died in Washington, Sept. 4, aged 50. His wife, Dr. Anita Newcome McGee was the only woman holding a commission in the U. S. Army. Dr. McGee left his body to Dr. Edward A. Spitzka, of Jefferson Medical College. His brain weighed 1410 grams, slightly above the average. We had met Dr. McGee a number of times in connection with archæologic matters and the mutual lack of front names immediately established a tie that developed into a warm personal feeling, in addition to the respect due to his scientific attainments.

